# Qualitative testing of potential modifications to the EQ-HWB EMV2.1 in adults with mobility problems in the United Kingdom

**DOI:** 10.1186/s12955-026-02577-x

**Published:** 2026-07-07

**Authors:** Aisha Moolla, Jill Carlton, Tessa Peasgood

**Affiliations:** Sheffield Centre for Health and Related Research (SCHARR), School of Medicine and Population Health, University of Sheffield, Sheffield, UK

**Keywords:** EQ-HWB-9, Content validity, Modifications, Health and wellbeing, Quality of life, Cognitive interviews, Qualitative, Mobility problems

## Abstract

**Background:**

The EQ-Health and Wellbeing (EQ-HWB) is a generic measure assessing the impact of health, social care, and informal caregiving on quality of life. The 9-item short form, the EQ-HWB-9 v1.1 (experimental, 2022), underwent international validation, with subsequent modifications to item wording, response options, layout, and item order resulting in the EQ-HWB EMV2.1 (experimental modified, 2025). This study aimed to assess the content validity of this version in individuals with mobility problems.

**Methods:**

Twelve adults in the UK with self-reported health conditions and mobility problems participated in semi-structured interviews. Participants described their health experiences, reviewed each EQ-HWB EMV2.1 item, and compared potential further modifications to the ‘mobility’ item wording and ‘cognition’ response options. Comprehension, relevance, comprehensiveness and preferences were explored using verbal probing. Interviews were transcribed and analysed using framework analysis, guided by content validity domains, with double-coding for 25% of transcripts to ensure alignment.

**Results:**

Participants (age 35–79 years) reported 35 health conditions. Eight items were understood as intended by all participants. The ‘mobility’ item presented varied interpretations (home-based limitations, daily activities, or aid use, n = 4), highlighting the need for an illustrative example, though no single wording was universally preferred. For ‘cognition’, participants understood both frequency and difficulty response scales; preferences were split, indicating no required modifications. Some overlap between items was noted (‘mobility’-‘activities’, ‘cognition’-‘exhaustion’/’anxiety’/’pain’), but participants did not perceive redundancy and valued separate items for nuanced measurement. Most items were considered relevant, and participants suggested additional content on medical management, support, mental health impact, and free-text contextualisation. The seven-day recall period and instructions were generally well understood.

**Conclusion:**

The EQ-HWB EMV2.1 demonstrates good content validity in a sample of patients with mobility problems, with most items comprehensible and relevant. The ‘mobility’ item would benefit from an example to reduce interpretive variability, while ‘cognition’ response options are appropriate. Some item overlap is expected in complex health populations, yet distinctions between items are meaningful. Participants highlighted the importance of healthcare experiences in quality-of-life assessment. These results inform instrument refinement, but final changes should integrate broader evidence from global validation studies across other populations and languages.

**Supplementary Information:**

The online version contains supplementary material available at https://doi.org/10.1186/s12955-026-02577-x.

## Background

The EQ-Health and Wellbeing (EQ-HWB) is a generic measure designed to assess the impact of health, social care and informal caregiving on quality of life [1]. The 25-item EQ-HWB and its 9-item short form (EQ-HWB-9) were developed through the EQALY project and tested for face validity and psychometric properties across six countries, after which they were designated ‘Experimental’ for further validation [2, 3].

International validation of the EQ-HWB-9 supported modifications to wording, response options, layout, and item order, resulting in the EQ-HWB-9 EMV2.1 (experimental modified, 2025) proposed by the EuroQol Working Group in September 2025 (Supplement 1). Five items (‘mobility’, ‘daily activities’, ‘cognition’, ‘control’, and ‘pain’) received slight additional revision at this stage to remove ambiguities identified in the validation studies (see Supplement 1). Here we aimed to confirm that these revisions improved item comprehension. For the 'mobility' item, conflicting evidence from multiple studies had been found relating to the use of aids. Accordingly, alternative wordings for this text were tested within our study. The primary objectives of this study were to assess the comprehensibility of the EQ-HWB EMV2.1 among those with mobility issues and to explore participants’ perceptions, preferences, and perceived differences between alternative item wordings. Secondary objectives focused on evaluating the relevance and comprehensiveness of the measure.

## Methods

### Participants

Adults (18 + years) residing in the UK with a self-reported health condition and mobility problem were recruited via Prolific (prolific.com). Participants were reimbursed £15 for participating in a 45-minute semi-structured interview over Google Meet. Ethical approval was granted by the University of Sheffield, reference 064729.

Following COSMIN (COnsensus-based Standards for the selection of health Measurement INstruments) guidelines recommending at least seven participants, a sample of 12 was considered sufficient [4]. In keeping with Standards for Reporting Qualitative Research [5], reflective considerations are also discussed (Supplement 2).

### Interview process

Participants provided consent online and completed interviews guided by a COSMIN-based topic guide (Supplement 3) [6]. They described their health experiences, reviewed EQ-HWB EMV2.1 items with verbal probing [8], and evaluated item modifications and overall comprehensiveness. Participants also completed a brief demographic questionnaire via Qualtrics covering their health, life satisfaction, education, and employment status.

All interviews were conducted by a trained researcher (medical doctor and health economist). The research team met regularly during data collection to monitor progress and address emerging issues. After the first three interviews, the protocol was amended to vary the order of item modifications to assess potential ordering effects on participant responses.

### Analysis

Interviews were transcribed verbatim, checked and analysed using framework analysis, including coding, charting in Excel, and mapping for synthesis [9]. Coding was guided by a pre-defined content validity framework (Supplement 4), and 25% of transcripts were double-coded for alignment and discrepancies resolved through team discussion.

## Results

### Participants

Twelve participants (35–79 years, 75% female) reported 35 health conditions (Table S1).

### Content validity

#### Comprehensibility

Eight items (‘activities’, ‘exhausted’, ‘lonely’, ‘thinking’, ‘anxious’, ‘sad/depressed’, ’control’ and ‘pain’) were understood as intended by all participants (Table S2).

**Fig. 1:**
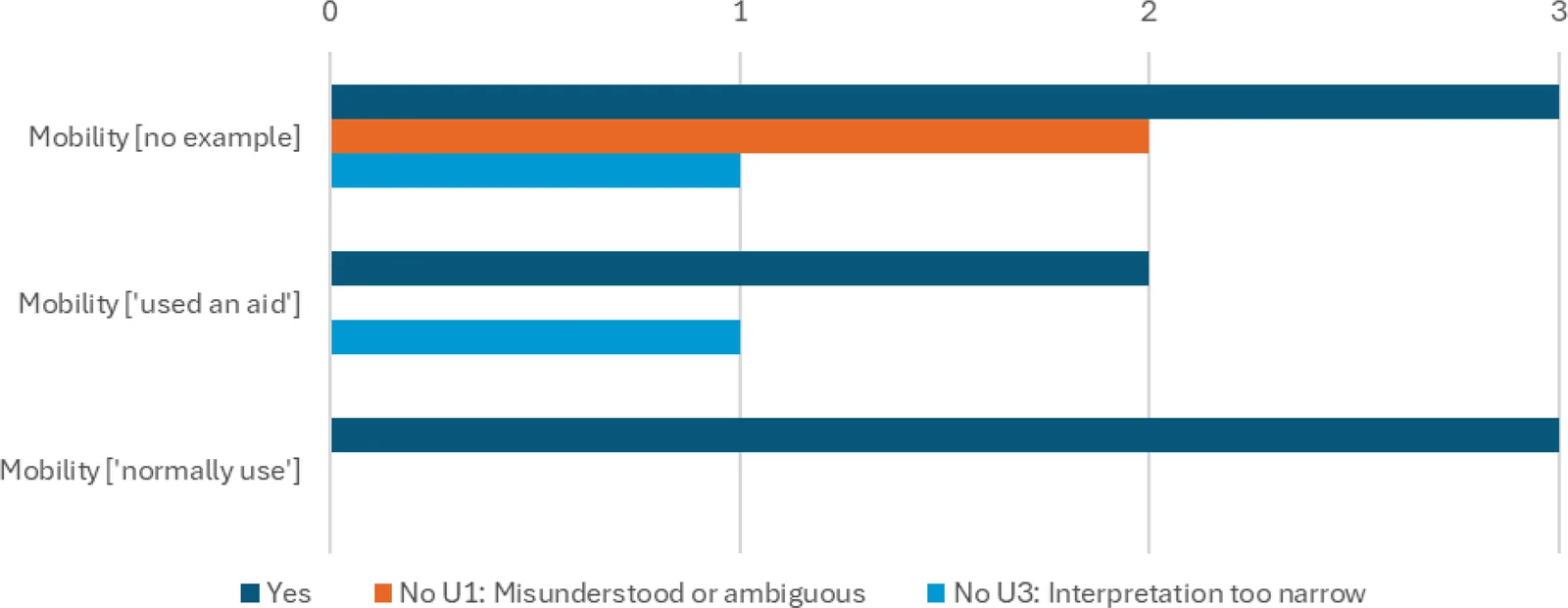
Number of interviews demonstrating understanding as intended for each version of the ‘mobility’ item. U1: Respondent interprets the item differently than what was intended by the developers. U3: Respondent focuses on one aspect of the construct or are unsure about the focus of the item

A version of the ‘mobility’ item without examples (“How much difficulty did you have getting around inside and outside?) was presented to six participants, followed by the ‘used’ version (“How much difficulty did you have getting around inside and outside? *If you used a mobility aid (such as a wheelchair or walking frame/walker)*,* please think about how difficult it was for you to get around when using the aid*”) (*n* = 3) and the ‘normally use’ version (“How much difficulty did you have getting around inside and outside? *If you normally use a mobility aid (such as a wheelchair or walking frame/walker)*,* please think about how difficult it is for you when using the aid*”) *(n* = 3) (Fig. 1). The item posed challenges for four participants. One participant interpreted it *[‘no example’]* as referring only to mobility limitations at home, stating, *“To me*,* that’s homebased. So*,* either in the house or in the garden”* *(08 F 60-)*. Another participant interpreted it *[‘no example’]* to be referring to daily activities, stating, *“…moving furniture around so I can clean up and clean the area.”* *(10 F 60-)*. The other two participants [shown ‘no example’ and ‘used’] understood the item to be asking whether or not aids were used, e.g., “*So*,* you’re kind of like asking*,* when I’m at home*,* do I need any support? So*,* can I walk around my house fine or do I need some assistance like a walking stick or wheelchair or just need some help?*” *(11 F 60-)*.

### Relevance

**Fig. 2:**
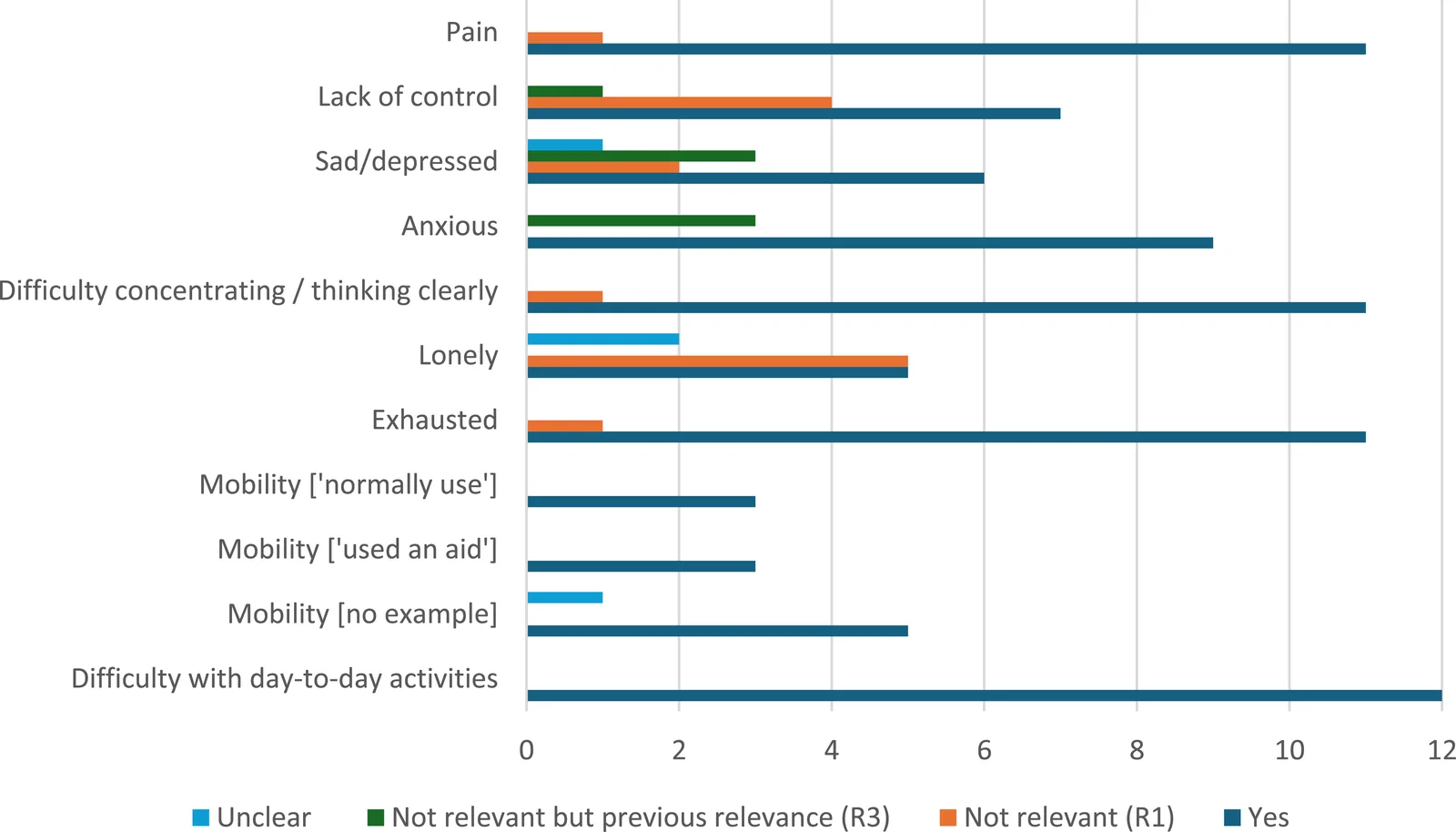
Number of interviews demonstrating relevance for each item. R1: Respondent reports that a question is irrelevant/inappropriate and should not be asked. R3: Respondent reports that a question is currently irrelevant/ inappropriate but was relevant at an earlier point in time

The majority of participants indicated relevance across all items. All items other than ‘activities’ and ‘mobility’ had at least one participant who said the question was not relevant, with five participants saying this was the case for ‘loneliness’, ‘sadness/depression’ and ‘control’ respectively (Table S3, Fig. 2).

An overlap between items was observed for ‘activities’, and ‘mobility’, ‘exhaustion’, and ‘pain’, with participants indicating an interplay between these items, e.g. *“[‘activities’ are affected by] how physically tired I am. How much energy I have. whether I’m in pain or not and how much that affects what I can do.” (01 M 60+).* Similarly, there was an overlap between ‘cognition’ and ‘exhaustion’, ‘anxiety’, and ‘pain’, e.g., *“I think that sometimes the pain can be so great that is difficult to concentrate and think clearly.” (09 F 60-)*.

### Preferences for potential modifications of the EQ-HWB-9

The reasons participants gave for their preferences were grouped into three main themes: ‘Appropriateness’, ‘Readability’ and ‘Distressing or unpleasantness’. Detailed summaries and supportive quotes can be found in Supplementary Tables S4 and S5.

#### Mobility modifications

Most participants (*n *= 10) perceived differences in meaning between versions of the ‘mobility’ item. Five [first shown ‘no example’ (*n* = 3) or ‘used’ (*n* = 2)] indicated that all versions were different. The ‘no example’ version was seen to alter the implied baseline for aid use. Additionally, ‘normally use’ and ‘used’ reflected chronic versus acute use of aids, e.g. *“So once you use the thing [aid]*,* that [‘normally use’/’used’] kind of equalizes you in the terms of the question with someone that doesn’t use one. Then how would you rate it?…. I think a lot of people would put ‘unable’ if they perhaps use a wheelchair [‘no example’]… I think that [‘used’] could be more relevant for somebody who perhaps has a temporary physical issue…they would probably write ‘a lot of difficulty’ if it didn’t say ‘if you used’ rather than ‘if you normally use’” (07 F 60-).* Four participants [first shown ‘no example’ (*n* = 3) or ‘normally use’ (*n* = 1)] saw ‘used’ and ‘normally use’ versions as similar, both indicating difficulty beyond the regular use of aids, while the ‘no example’ version referred to whether or not aids were used. One participant [first shown ‘normally use’ (*n* = 1)] considered ‘normally use’ and ‘no example’ equivalent, both reflecting difficulty beyond the regular use of aids, and distinct from ‘used’, which indicated whether or not aids were used.

Preferences for item wording varied among participants. Three participants preferred ‘no example’ compared to eight who preferred the inclusion of an example, citing improved readability [*n* = 2; first shown ‘used’ (*n* = 1) or ‘normally use’ (*n* = 1)] or a personal preference (*n* = 1; first shown ‘used’). Five preferred ‘used’ for its inclusivity of acute and chronic aid use [*n* = 3; first shown ‘no example’ (*n* = 2) or ‘normally use’ (*n* = 1)], less formal tone (*n* = 1; first shown ‘normally use’), and alignment with the recall period (*n* = 1; first shown ‘used’). Three preferred ‘normally use’ for similar inclusivity (*n* = 2, first shown ‘no example’) and for being less emotive (*n* = 1, first shown ‘no example’). One participant (first shown ‘no example’) preferred combining ‘used’ and ‘normally use’. Overall, those first shown ‘no example’ always preferred a version inclusive of an example.

#### Response scale modification for the cognition item

Most participants perceived differences between the frequency and difficulty scales for ‘cognition’ (*n* = 7), primarily noting that the severity implied by the difficulty scale may not align with frequency (*n* = 6) or that difficulty was considered a more impactful metric (*n* = 1). Five participants viewed the scales as equivalent.

Six participants favoured the difficulty scale, citing its appropriateness as a more important measure (*n* = 3) and greater ease of response (*n* = 3). Five participants preferred the frequency scale, with one emphasising frequency as more important and four noting greater ease of response. One participant suggested using separate questions depending on the intended outcome.

### Comprehensiveness

Only two participants viewed the measure as fully comprehensive (Table S6). Ten identified missing information such as receiving adequate medical management (*n* = 4), support (*n* = 2), emphasis on the effect of health conditions on mental health (*n* = 2), and the need for free text for contextualisation (*n* = 2).

Two participants suggested merging ‘anxiety’ with ‘sad/depressed’ or ‘loneliness’ with ‘sad/depressed’ and ‘control’, respectively (Table S7). Some participants found the seven-day recall period appropriate (*n* = 2), while others preferred shorter recall for clarity or to capture peak experiences (*n* = 2) (Table S8). Issues with response options were reported (*n* = 3), including unclear wording and difficulty capturing both intensity and frequency. Instructions were considered well written by two participants, but one participant found instructions *“Think about your health*,* feelings and whether you were able to do your activities or have them done for you as you wanted”* repetitive and another found *“able to do your activities or have them done for you as you wanted”* confusing. Six participants viewed the questionnaire positively for relevance, clarity, and comprehensiveness when prompted to provide general feedback.

## Discussion

This study highlights the challenges of achieving precise and universally interpretable wording, particularly for the ‘mobility’ item, where participants’ understanding varied between home-based limitations, daily activities, and the use of aids (*n* = 4), primarily among those who were first shown the ‘no example’ version (*n* = 3). These differences suggest that wording alone cannot resolve all comprehension issues, emphasising the need to anchor item design in theoretical definitions rather than individual preference. While no single wording was universally preferred, participants consistently indicated that including some form of example would be helpful to clarify the intended meaning of the ‘mobility’ item. In particular, those who first received ‘no example’ indicated a preference for some form of example. Order effects may have influenced responses, especially given the small sample. For the current EMV2.1 version, four additional items underwent slight wording changes. This study confirms that comprehension of these four items remains high. For the unchanged items (‘exhausted’, ‘lonely’, ‘anxious’, ‘sad/depressed’), these findings are confirmatory and consistent with prior evidence.

For ‘cognition’, although preferences between frequency and difficulty scales were split, participants demonstrated accurate understanding of the existing wording, indicating that no modifications are necessary.

Overlap between items was observed, notably between ‘mobility’ and ‘activities’, and between ‘cognition’ and ‘exhaustion’, ‘anxiety’ or ‘pain’, reflecting the interconnected nature of participants’ health conditions. According to COSMIN guidance, excessive overlap between items can reduce content validity, affect structural validity, and suggest improper item generation [4]. However, in this study, participants did not perceive overlapping items as redundant; instead, they appreciated the separate measurement of related constructs, demonstrating accurate understanding of each item. This supports the inclusion of distinct items to capture nuanced aspects of participants’ experiences, while acknowledging that some correlation between related items is expected in populations with complex or multiple conditions [6].

Participants frequently indicated the importance of capturing aspects of medical care, suggesting that satisfaction with healthcare is a relevant component of quality of life for this patient group. The EQ‑HWB‑9, however, was designed for situations where brevity is best. The missing content identified by participants represents constructs that were either intentionally omitted to preserve this (e.g., details of medical care) or fall outside the measure’s intended scope (e.g., satisfaction with healthcare). Therefore, adding these items to the EQ‑HWB‑9 is not recommended. Researchers who require additional long-form content should consider using supplementary tools designed to capture healthcare experience and care quality. Tools such as the Q-LES-Q (Quality of Life Enjoyment and Satisfaction Questionnaire) [7], FAMCARE-2 [8], and QUEST (Quality of End-of-Life Care and Satisfaction with Treatment) [9] explicitly measure satisfaction with treatment or care quality, highlighting the potential value of including similar considerations in and/or alongside general quality-of-life instruments.

This study has some limitations. Given the small sample of 12, genuine preference cannot be separated from order effects related to the ‘mobility’ item. Thus, while an illustrative example is helpful for removing potential ambiguity for this sample, a definitive recommendation regarding ‘used’ over ‘normally use’ (or vice versa) cannot be made. Larger studies are needed to resolve this. Further, a limitation of this study is the sample’s limited ethnic diversity, gender imbalance (75% female), and high education level (58% bachelor’s degree or above) which may affect transferability to more diverse populations. With split preferences (6 vs. 5) and accurate understanding of both scales of the ‘cognition’ item, qualitative evidence was insufficient to select a response scale. Additional quantitative evidence (e.g., test-retest reliability, known-groups validity, differential item functioning, responsiveness to change, or anchoring to a global cognition rating) could support findings. Cross-country comparability and existing conventions may also be considered as part of a broader evaluation.

This study’s sample was purposively selected to include adults with mobility problems, as the ‘mobility’ item had undergone the most significant change following prior validation work. This design choice enhances the relevance of findings for that specific item: individuals without mobility limitations would likely respond ‘no difficulty’ regardless of wording variations, making comprehension variability less consequential. For those with mobility impairments, who represent the primary target population for this item, participants found that an illustrative example is beneficial for supporting consistency of interpretation and is directly transferable across clinical settings. For the remaining four items with changes, there was high comprehension among participants.

## Conclusions

The EQ-HWB EMV2.1 demonstrates good content validity in British adults with mobility problems. Most items were understood as intended and relevant to participants. For the ‘mobility item’, which was found to have significant comprehension issues, an illustrative example was found to be beneficial for reducing interpretive variability in this target population. The ‘cognition’ item response scale is well understood and requires no change. Some item overlap is inevitable given participants’ complex health conditions, but distinctions remain meaningful, supporting separate measurement of related constructs. These findings provide guidance for refining the instrument. However, adjustments should be informed by broader evidence from global validation studies in other populations (e.g., general population, carers, social care users) and other languages.

## Supplementary Information

Below is the link to the electronic supplementary material.

Supplementary Material 1

## Data Availability

The datasets generated during the current study are not publicly available due to the data containing information that could compromise the privacy of research participants. All data used to support the findings reported are included in the supplementary material.

## Abbreviations

EQ-HWB: EQ-Health and Wellbeing
COSMIN: COnsensus-based Standards for the selection of health Measurement INstruments
Q-LES-Q: Quality of Life Enjoyment and Satisfaction Questionnaire
QUEST: Quality of End-of-Life Care and Satisfaction with Treatment
