# Supplementary material for: Qualitative testing of potential modifications to the EQ-HWB EMV2.1 in adults with mobility problems in the United Kingdom
Source: Health Qual Life Outcomes. 2026 Jul 7;24:110. doi: 10.1186/s12955-026-02577-x (PMC13546503; doi:10.1186/s12955-026-02577-x)
Supplement: Supplementary file 1 [file 12955_2026_2577_MOESM1_ESM.docx]

**Supplementary materials:** **Qualitative testing of potential modifications to the EQ-HWB-9 EMV2.1 in the United Kingdom**

Contents

[Supplement 1: EQ-HWB-9 EMV2.1 Proposed Modifications for Testing	2](#_Toc225336263)

[Supplement 2: Reflective considerations	4](#_Toc225336264)

[Supplement 3: Topic Guide	4](#_Toc225336265)

[Supplement 4: Coding framework	9](#_Toc225336266)

[Supplement 5: Tables	10](#_Toc225336267)

[Table S1: Description of Participants (n=12)	10](#_Toc225336268)

[Table S2 Quotes for item understanding in EQ-HWB-9	12](#_Toc225336269)

[Table S3 Quotes for item relevance for EQ-HWB-9	16](#_Toc225336270)

[Table S4a: Summary of the themes for perceived difference between version of the item Get around: ‘no example’ or ‘used’ or ‘normally use’	24](#_Toc225336271)

[Table S4b: Themes and quotes supporting preferences for modification of the item Get around: ‘no example’ or ‘used’ or ‘normally use’	24](#_Toc225336272)

[Table S5a: Summary of themes for perceived differences for scale of the item Concentrating/Thinking clearly: Frequency scale vs Difficulty scale	27](#_Toc225336273)

[Table S5b: Summary of themes and quotes on preferences for response options ‘moderate’ vs ‘some’	27](#_Toc225336274)

[Table S6 Quotes for comprehensiveness (missing items) of EQ-HWB-9	28](#_Toc225336275)

[Table S7 Quotes for comprehensiveness (redundant) of EQ-HWB-9	30](#_Toc225336276)

[Table S8 Quotes for additional commentary of the EQ-HWB-9	31](#_Toc225336277)

## Supplement 1: EQ-HWB-9 EMV2.1 Proposed Modifications for Testing

Where items are marked *, this notes wording that was modified from the EMV2.0 to the EMV2.1 version.

SAMPLE

| **Instructions for completion** | **Amend (Y/N)** | **Proposed further modifications for testing** |
| --- | --- | --- |
| These questions are about your health and wellbeing in the last 7 days. | N |  |
| Think about your health, feelings and whether you were able to do your activities or have them done for you as you wanted.* | N |  |
| Please choose one answer for each question. | N |  |
| **EQ-HWB-9 items (Response level D/F/S)** | **Amend (Y/N)** | **Proposed further modifications for testing** |
| How much difficulty did you have doing day-to-day activities?* | N |  |
| How much difficulty did you have getting around inside and outside? (D)*  SAMPLE | Y | Proposed possible alternative additional wording, provided in italics after the item:   1. *If you normally use a mobility aid (such as a wheelchair or walking frame/walker), please think about how difficult it is for you when using the aid* 2. *If you used a mobility aid (such as a wheelchair or walking frame/walker), please think about how difficult it was for you to get around when using the aid.* |
| *In the last 7 days, did you:* |  |  |
| feel exhausted? (F) | N |  |
| feel lonely? (F) | N |  |
| have difficulty thinking clearly? (F)  *Meaning you had difficulty thinking, concentrating or remembering** | Y | Propose possible change of response options from **‘frequency’ to ‘difficulty’**. |
| feel anxious? (F) | N |  |
| feel sad or depressed? (F) | N |  |
| feel you lacked control over your day-to-day life?  *Meaning you lacked choice over what you did or how things were done for you* (F)* | N |  |
| In the last 7 days, did you **have physical pain**?  no / mild / moderate / severe / very severe physical pain? (S)*  SAMPLE | N |  |
| **Response levels** | **Amend (Y/N)** | **Proposed modifications** |
| Difficulty (D): no / slight / moderate / a lot of difficulty / unable | N |  |
| Frequency (F): none of the time /a little of the time / sometimes / often / most or all of the time | N |  |
| Severity (S): no / mild / moderate / severe / very severe | N |  |

***© EuroQol Research Foundation. EQ-HWB^TM^ is a trademark of the EuroQol Research Foundation. This is a modified EQ-HWB. Reproduced by permission of EuroQol Research Foundation. Reproduction of this version is not allowed. For reproduction, use or modification of the EQ-HWB (any version), please register your study by using the online EQ registration page:*** [***www.euroqol.org***](http://www.euroqol.org)

## Supplement 2: Reflective considerations

The interviewers were conducted and coded by one young female researchers (AM) who has had prior involvement in conducting interviews related to the validation of the EQ-HWB, but has not been involved in the development of the EQ-HWB. It is possible that participants of a different gender may have felt less comfortable disclosing information relating to their health and wellbeing. However, the style of interview relying mostly upon verbal probing and interpretation of items is less personal than a predominantly ‘think-aloud’ approach.

The other members of the research team (TP and JC) were involved in the EQ-HWB development and may therefore have a vested interest in demonstrating that the instrument is performing well. However, preferences between original versions and potential modifications, which form a substantial component of the research, are neutral in this respect.

## The interviews were conducted by a medical doctor and health economist (AM). This dual background supported engagement with participants on both health and wellbeing concepts, while also introducing the possibility of interpreting responses through professional and technical lenses. All interviewees were adults with medical conditions, which facilitated rapport but also introduced differences in lived experience. Within the research team, the interviewer was the least experienced member but had autonomy to adapt the interview process and analysis. This reduced the influence of senior colleagues’ perspectives or biases and helped ensure that participants’ voices remained central.

## Supplement 3: Topic Guide

**Aims and Objectives**

- To provide evidence to support decision-making on the final content for the EQ-HWB-9
- To test wording of final items, including the examples provided in brackets and the response options
- To test understanding (i.e., comprehensibility) of the modified items
- To test relevance of the modified items. This will include whether the item is relevant and important for them currently in the context of being someone with a health condition.

**In advance of the Interview**

- Please see the intended item meaning for each item in the document
- Looking at the EQ-HWB intended meaning of items document will allow for a better understanding of how we hope responders will interpret the items. When a participant then talks about what they think about a draft item, it will be apparent to you during the session whether the item has been understood as intended.
- Prepare some useful general probes, for example:
  - *Can we just think about whether this is relevant for you?*

**During the Interview**

- When first discussing the draft items, do not state the name of question, neither should you mention any of the examples. Instead, consider saying “*look at the question number 1*” (for example)
- Probe participants for examples, i.e., to provide context to their views, experiences, and opinions.
- It is important to elicit data for each component of an item (e.g. understanding and relevance).
- A distress protocol [not included here] will be initiated should participants become upset
- Ensure recording occurs in line with ethical approvals
- For online interviews remind participants that it is important to have their camera on throughout the interview.

**Key:** Italics in blue denotes words for interviewer to use.

**Introductions and welcome**

- Introduce interviewer and their role
- Remind participants that the session is being recorded.
- Remind participants that if there is something that they do not want to discuss, that is ok. For example, you can tell them they can simply say they would prefer to pass on that question.

**Warm up and focusing the future discussion**

- To help frame the discussion to ‘health’ or ‘carer’ or ‘social care’ related aspects of quality of life ask the participant to start thinking about how the relevant aspect impacts on their life

*Before we get started on the questionnaire could you tell me a little bit about how your health impacts on your life.*

- This will be kept short – it’s really just to warm up the participant

*Thank you.*

*We’ll move on to look at the question that has been developed to measure the quality of life of adults with health conditions or people with additional needs, and for people who are caring for people with additional needs.*

*We would like to find out what people think about different ways that we can ask the questions.*

*There’s no right or wrong answer with anything I ask you today. We are really interested to hear your views and about your experiences.*

***(U=understanding, R=Relevance)**

| **Component to be tested** | **Wording and probes** | | **What are we testing?** |
| --- | --- | --- | --- |
| Completion instructions | *Take a look at the questionnaire, specifically at the instructions on the top of page number 1, which start with the words “These questions are about your health and wellbeing in the last 7 days”. Could you explain, in their own words, what you feel the instructions are asking you to do.*   - If issues with the recall period of 7 days are raised, encourage participant to provide examples (e.g., provide context as to whether the time period is considered suitable).   . | | U |
| Question 1-9 | *Now look at question 1. What does this question mean to you?*   - Encourage participants to answer in a way that can provide evidence to support understanding the item concept. i.e., encourage examples (e.g., context). - Probe if there are additional words that could be included in the example. i.e., encourage examples (e.g., context). - Probe if any of the words and examples are unclear and/or redundant. i.e., encourage examples (e.g., context). - Additional probes that could be considered if participants were not able to fully describe what the item meant:   - *‘What would an example of ‘moderate difficulty* [insert question] *be like? Could you give an example?’* | | U |
|  | *We’d like to know whether this question would be relevant to ask when thinking about how your health impacts on your life. When you look at this question, what sort of things are you thinking about, with respect to your own health or caring role?* | | R |
| VERSION 2 & COMPARISONS | | | |
| Question 2 *(In the last 7 days, how much difficulty did you have getting around inside or outside? If you normally use a mobility aid (such as a wheelchair or walking frame/walker), please answer based on how difficult it is for you when using the aid.)* | *Can you have a look at this slightly different way of asking the question – from your point of view does it seem to be asking anything different when it’s asked like that?*   - *Probe about the any alternatives to the description of aids*   *Would your response be the same or different for each of these questions?*  *Which of these versions do you like best?*  *Probe as to why* | | U&R |
| Question 5 (*In the last 7 days, did you have difficulty thinking clearly? Meaning you had difficulty thinking, concentrating or remembering)* | *You’ll see in the first questionnaire the response answers use the response ‘no difficulty, slight difficulty, etc’ rather than ‘none of the time, a little of the time, etc’. Do you think these are the same or different?*  *Would your response be the same or different for each of these questions?*  *Which of these versions do you like best?*  *Probe as to why* | | U&R |
| **Understanding** | | | |
| Completion instructions | *Now that we’ve gone through the questionnaire, take a look at the instructions on the top of page number 1 again, which start with the words “These questions are about your health and wellbeing in the last 7 days”. Do you feel that they are clear?* | |  |
| COMPREHENSIVENESS OF MODIFIED VERSION | | | |
| Wrap up (considering the whole EQ-HWB-9 questionnaire) | *Having looked at all the questions, when you think about the impact of [health/your caring role] on your life is there anything missing? Are there any other questions that you think we should we have included that are not currently there?*   - Establish whether any new suggestions relates to any specific health condition or need - Encourage participant(s) to give examples (i.e., context). | Comprehensiveness | |
|  | *This is designed to be a short questionnaire - is there anything in there that you don't think is important or where you think we have questions that are a bit repetitive?* | Redundancy / Lack of relevance | |
|  | *Finally, do you have any other comments or suggestions to feedback on about the questionnaire or any of the questions we’ve looked at today?* | Comprehensiveness | |

**Closing of the interview**

**Collect demographics**

Explain that this is the end of the discussion.

Thank the participants for their time – provide participants with an opportunity for a debrief discussion on how answering these questions has made them feel.

Explain when participants can expect to receive their reimbursement.

## Supplement 4: Coding framework

| **Understanding or interpretation correct including value of examples** | Respondent understands and interprets the item correctly |
| --- | --- |
| U1Wrong or ambiguous interpretation | Respondent interprets the item differently than what was intended by the developers |
| U2 Difficulty interpreting/ odd wording | Respondent comments that they are unfamiliar with terms/phrases or struggles with a complicated structure. Respondent finds terms/phrases unusual or odd. |
| U3 Narrow interpretation | Respondent focuses on one aspect of the construct or are unsure about the focus of the item |
| U4 Word means a different thing in translation* | Word used in translation does not match the intended meaning |
| U5 Different answers for different aspects of items | Respondent has difficulty with double-barrelled items |
| U6 Issues with examples | Respondent has difficulty with examples used and interpret the item incorrectly as a result |
| Unclear | Unclear if respondent understands |
| **Relevance of item** |  |
| R1 Item not relevant | Respondent reports that a question is irrelevant/ inappropriate and should not be asked. This should be contextualised appropriately i.e. if a mixed population is used it should be clear which group thinks this e.g. carers |
| R2 Respondent says item not relevant because they misunderstood item | Respondent reports the item is not relevant, but they misunderstood the question therefore relevance is not clear. |
| R3 Item previously relevant | Respondent reports that a question is currently irrelevant/ inappropriate but was relevant at an earlier point in time. |
| Unclear |  |

*Note that concerns over translation are not relevant in the UK context, but the same core coding framework was applied to all related international studies.

## Supplement 5: Tables

## Table S1: Description of Participants (n=12)

|  | **N** | **%** |
| --- | --- | --- |
| **Female** | 9 | 75 |
| **Age:** Mean (SD), range | 56 (SD 14.21), 35-79 years | |
| **Ethnicity**: White | 11 | 92 |
| **Education**: Bachelors degree or above | 7 | 58 |
| **Employment**: In employment/self-employed | 7 | 58 |
| **Long term condition** (*Do you have any long-standing physical or mental impairment, illness or disability which has been diagnosed by a doctor? By 'long-standing' we mean anything that has troubled you over a period of at least 12 months or that is likely to trouble you over a period of at least 12 months)* | 12 | 100 |
| **Informal carer** (“*Is there anyone (family or friend), either living with you or not living with you, who is sick, disabled or elderly whom you look after or give special help? Please exclude paid caring work.*”) | 1 | 8 |
| **Social care users** (*Have you used social care services or support for yourself over the last 6 months (e.g. home care, home help, Personal Assistant, day-centre, residential care or nursing care home)?*”. | 0 | 0 |
| **Health conditions (patients may report more than 1)** |  |  |
| Asthma | 6 | 17 |
| Arthritis | 4 | 11 |
| Fibromyalgia | 4 | 11 |
| Coronary heart disease | 1 | 3 |
| Angina | 1 | 3 |
| Diabetes | 3 | 9 |
| Chronic back pain | 1 | 3 |
| Heart attack or myocardial infarction | 1 | 3 |
| High blood pressure/hypertension | 5 | 14 |
| Mental health condition | 2 | 6 |
| Thyroid disease | 2 | 6 |
| Rheumatoid arthritis | 1 | 3 |
| Fracture | 1 | 3 |
| Chronic fatigue syndrome | 1 | 3 |
| Collapsed lung | 1 | 3 |
| Other long standing or chronic condition | 1 | 3 |
| **Duration of the longest condition reported (mean)** | 19.08 years |  |
| **Carers hours per week spent caring** |  |  |
| 1-19 | 0 |  |
| 20-49 | 1 | 100 |
| 50+ | 0 |  |
| **Health overall** |  |  |
| Very good | 0 | 0 |
| Good | 1 | 8 |
| Fair | 5 | 42 |
| Bad | 5 | 42 |
| Very bad | 1 | 8 |
|  |  |  |
| Life satisfaction (0-10), Mean (SD) | 6.14 (SD 1.95) | |

*Note that all participants reported having “long-standing physical or mental impairment, illness or disability which has been diagnosed by a doctor?”. It is therefore assumed that these participants have a disability or impairment they do not consider to be a health condition.*

## Table S2 Quotes for item understanding in EQ-HWB-9

(see Supplement 3 above for an explanation of the codes applied)

| **Item** | **Demonstrating understanding aligned to intended meaning** | **Demonstrating concern with understanding** |
| --- | --- | --- |
| Activities | - *“how difficult it's been for me to carry out day-to-day activities like dressing, cooking, household chores, doing work.” (01 M 60+)* - *“…that would be the basic functioning things such as being able to cook and clean...I would think something like needing help to go to the shops. perhaps, to physically get to the shops and maybe when you're in the shops as well. perhaps not always being able to manage to cook a full meal and other times relying on ready meals.” (07 F 60-)* - *“what it's talking about there is the kind of things like, is your mobility good enough for you to say go to the toilet unassisted? Can you make yourself a meal? can you go to the shop on your own?” (06 F 60+)* | 100% understand  Overlap observed with mobility/exhaustion/pain (see relevance) |
| Mobility | - *“Are they able to get up off a chair, walk through to the kitchen? Can they go to the bathroom? If we've got stairs in our house, were they able to get up and down the stairs easily, transporting yourself outside again? did they have difficulties? possibly as simple as stepping off a curb to crossing roads.” (02 M 60+)* - *"I can go to shops nearby but I can’t go far away. When thinking inside the house I would think, is it impacted the amount of what I can move about. So for example my house doesn't have bathroom upstairs. Is it all right for me to go up and downstairs as many time as I need inside the house. so if I say many times I couldn’t to going to the toilet so I go less times upstairs that would be moderate difficulty" (09 F 60-)* | - U1 (Wrong understanding):   “*inside, moving furniture around so I can clean up and clean the area. So being able to move the seti or move my desk in order so I can just clean up and hoover up and stuff. I'd find that moderate difficulty to really a lot of difficulty because I can't move the sofa nowadays… being able to take the vacuum cleaner upstairs so I can hoover upstairs. That I would find a lot of difficulty."* (10 F 60-; shown ‘no example’)   - U1: (Issues arose because of the examples):   *"I would think inside they might use disability aids perhaps like handrails or perch stall, that kind of thing. or need a ramp. Getting around outside for moderate, I would think would definitely be ambulatory wheelchair use or mobility scooter use. I would think if it's moderate difficulty, it's going to be more than using a walking stick. Perhaps using crutches because that's both sides but I would think it would need an aid."* (07 F 60-, shown ‘no example’)   - U3 (Narrow interpretation of mobility in the home only): *“To me, that's homebased. So, either in the house or in the garden, that kind of thing. Going out to put the washing out as opposed to going out...Whether there are stairs or steps in the home that they have to navigate, how long it takes them to move around, whether they're looking for things to hold on to or support themselves… if they're going into a garden whether they've got ramp access or something like that if they do have accessibility issues".* (08 F 60-, shown ‘no example’) - U6: (Issues arose because of the examples) *“So, you're kind of like asking when I'm at home do I need any support? So, can I walk around my house fine or do I need some assistance like a walking stick or wheelchair or just need some help? even in the kind of shower or anything.”* (11 F 60-, shown ‘used’) |
| Exhausted | - *“Exhausted to me is more than tired. it's gone. its extreme tiredness, fatigue beyond what can reasonably be foreseen from the activities that you might have done.” (08 F 60-)* - *""if you're absolutely wiped out and you're not capable of really doing a lot, feeling very tired and probably aching all over." (01 M 60+)* | 100% understand as expected |
| Lonely | - *'"it's not necessarily the amount of social interaction you have though. It's a case of whether you feel it's enough. but also you can have a lot of interactions and still feel lonely. so I think, it's just implying do you feel like you're on your own mentally or physically enough that it impacts you?" (07 F 60-)* - *"It means do you feel alone but that doesn't generally just cover being alone in itself. it also means that sometimes you're surrounded by people but you still feel alone. You don't feel seen. You don't seem like you have help enough. you don't seem like you have company or somebody you can count on." (09 F 60-)* | 100% understand as expected |
| Difficulty concentrating / thinking clearly | - *“This is like what I mentioned earlier in terms of brain fog, confusion, difficulty in concentrating….I’m really thick headed, I really battle to concentrate, I just feel totally lethargic.” (01 M 60+)* - *"So it is asking, how is my head? So am I able to concentrate on things? Am I able to enjoy normal shows that I like and concentrate reading a book or just generally thinking. sometimes when you can't think clearly you have brain fog where you can’t remember things well or you can't just… the process makes your brain so exhausted that you struggle to cope. You keep reading the same thing every.. always… constantly to try and get that in but you struggle with it" (09 F 60-)* | 100% understand as expected  Overlap observed with exhaustion/anxiety/pain (see relevance) |
| Anxious | - *“worried about how you're going to look, how you’re going to feel. It can also be anxiety about your actual medical condition and whether you can get any treatment for it and whether any doctor's going to listen to you and how long you're going to have it for. And it can really impact you because you know that a lot of the things, there's no treatment for it and it's really causes some stress” (05 F 60+)* - *"In terms of kind of like hyper vigilance, fearing the worst, particularly like when I'm trying to sleep at night as well… everything fires up then...I couldn't sleep because I was just so panicked. And it's really hard to calm down when you've got into a spiral like that...Sometimes, even other people…I just tend to avoid a lot of people. That makes that easier because other people are so unpredictable." (07 F 60-)* | 100% understand as expected |
| Sad/depressed | - *“I think again, are the medical problems or the ailments dragging your mood down…if you have got ongoing medical issues, you almost feel as if you're in a morass of ‘I can't get out of this. I can't escape and nothing's going right for me...Why is it me? why not somebody else? look at them, they're healthy’.” (02 M 60+)* - *“they'd be losing interest in things that you would normally find enjoyable. Not having any energy, not wanting to talk to people. not being bothered to dress properly or feed yourself properly or take care of yourself. feeling that there is no hope." (06 F 60+)* | 100% understand as expected |
| Lacked control | - *"people don't turn up at day centres sometimes and you wonder why. and it's because a relative hasn't got them dressed or hasn't given them the breakfast in time to be picked up and things like that. So yeah, they're lacking control in their day lives." (03 M 60+)* - *“I don't drive with all my tremors and whatever. So I have to rely on people a lot to take me places or to take me to doctors and hospitals and sometimes you just feel like you're not in control of your own life and sometimes you feel that your life is just revolved around doctors, pharmacies, physios… so definitely feel a lack of control like what I do is not in my head what I want to do." (11 F 60-)* | 100% understand as expected |
| Pain | - *“what I would call a stabbing pain. It could be a dull throbbing ache akin to toothache but in your joints or muscles wherever. Head pain is probably the best example. Someone suffering from migraines or whatever or just, the pounding headache as if your skull's going to explode, that sort of thing. so I think there's a gambit of the way physical pain can manifest itself.” (02 M 60+)* - *"So for example I have a moderate pain at the moment in my knee. It bothers me when I go up and down the stairs but it doesn't really impact my life that much. I just push it through power through it and it keeps going but it's always there. So it's moderate. It's not enough to stop me doing things, but it's enough to disturb me and make me constantly aware of it…basically the one that you get when you go to a hospital on a scale of 0 to 10, how high you would score your pain." (09 F 60- )* | 100% understand as expected |

*Each participant is represented by interview number (01-12), sex (F/M), and age group (60-, 60+) e.g. 04 M 60+*

*Where multiple quotes demonstrate understanding as aligned to the intended meaning only two are shown; for concerns with understanding all cases are shown.*

## Table S3 Quotes for item relevance for EQ-HWB-9

| **Item** | **Relevant** | **Not relevant** | **Relevance Unclear** |
| --- | --- | --- | --- |
| Activities | - *“how physically tired I am. How much energy I have. whether I'm in pain or not and how much that affects what I can do.” (01 M 60+)* - *“So immediately I just think about the challenge I have getting jobs done in the house. Having the energy and being physically able to deal with things as well, I am obviously classed as disabled and I have issues… sometimes I'm even unable to get out of bed. The pain is just too great. So it's definitely relevant, and it's also about concentration and focusing on things and whether or not I guess, if you're still working, whether or not you can sort of.. cuz it's not about having time off, is It's about being reliable. so if I looked at that question in those terms, I would say that I come moderately or a lot of difficulty...it's just going up and downstairs. It's being able to even use a hoover. I'm a bit clumsy. So, even sometimes I have pins and needles in my hands and I break things which drives me mad." (A03 M 60+)* | None judged not relevant | None judged unclear |
| Mobility | - *"I'm going to say moderate difficulty because at the moment, I don't know if its sciatica or its this muscle thing. I am having quite a lot of difficulty going upstairs actually. on the flat, absolutely fine…If I want to go upstairs, especially if I want to take a bag of laundry upstairs, it's a case of clinging onto the banisters and going up one step, it's difficult.” (06 F 60+)* - *“So we have stairs in the house. when I've come down in the morning, I tend not to go back upstairs until the end of the day." (08 F 60-)* | None judged not relevant | *“Cleaning up in the bathroom area. I find difficult. cleaning up outside, not necessarily any garden work, just cleaning up outside. just things like taking the bins out for it to be done weekly. I find difficult. if I was to go in the car and get shopping, I'd find it difficult to bring it back in. yeah.” (10 F 60-, shown ‘no example’)* |
| Exhausted | - *“when I'm asked do I feel exhausted, it's the fact that yes, I feel exhausted. And most of the time it's completely… I can't even say the word…was it [debilitating]...My head constantly has thoughts going around in my head. It's just… And that drives me mad, to” (04 F 60+)* - *"* *a lot of illness causes fatigue which is just generally extreme of exhausting, it's similar feeling. so is relevant. when I see this question, I think do I feel exhausted all the time and I do and it's an exhaustion that sleep can’t fix it. I don't see how can it be fixed because my sleep is deeply impacted by the pains. So what comes to my mind is that, do I have the same energy that I should have to do things or most of the time I struggle to even begin something" (09 F 60-)* | - *R1 "It's not one that… I would be quite happy to put none of the time." (03 M 60+)* | None judged unclear |
| Lonely | - *“But I've got a husband, but there are times when you can feel a bit lonely in the sense cuz it's so difficult to get my husband to understand sometimes what I'm going through.” (05 F 60+)* - *“when you have got chronic diseases, it can be lonely because people get fed up if you make plans and then at the last minute you have to cancel. They don't understand. and they think you're playing them around or you just don't want to be friends with them no more or you've got a better offer or sometimes when you go on about your diseases, people will get just fed up of listening to you...so it does drive people away, you don't mean to but you're just always in pain so it's really all you can think about and you become quite selfish because of that. So yeah, I think you can and get lonely." (11 F 60-)* | - *R1 “No, I don't feel I'm lonely. I've got a lovely wife and I've got family close by and I've got two children and I’ve got a grandkids. No, I've got plenty. I never feel that way.” (01 M 60+)* - *R1 “I'm happy in my own skin if I can put it that way as well. But I have the support of a very very caring family group round about me which I know not everyone does have.” (02 M 60+)* - *R 1 “I don’t. I'm lucky. I'm married. I've been married 58 years. Better get that right, 59 years. which is a long time. I've got four grandchildren, two of whom we see regularly, two of whom we keep in touch with by cheque book. But the two that we see regularly, they never let me feel lonely.” (03 M 60+)* - *R1 “I live on my own. I don't have any children. so you would think I'm going to say yes, but I'm going to say no...If I literally step out there, I will see 10 neighbours that I will chat on to about this that and the other. on Friday, I just go on my own to a social club.” (06 F 60+)* - *R1 "I live with my partner. He does a lot of the stuff for me when I can't do it, so not really relevant for me, but I can see why it would be relevant for other people." (12 F 60-)* | *“I'm thinking about the fact that I don't really get out much at all. And so I think by a lot of people's definition that would be lonely, but I also have a lot of anxiety and interactions with people. So in some ways it's also peaceful. so it's kind of a double-edged sword like that because it's both things.” (07 F 60-)*  *“I've restricted my friendship group, my involvement in activities. I've deliberately not got involved. that doesn't make me feel lonely, but sometimes I do feel I miss out." (08 F 60-)* |
| Difficulty concentrating / thinking clearly | - *“if I'm suffering from a fatigue episode, then that really applies to me...I mean when it comes over you, you really aren't really capable of thinking clearly.” (01 M 60+)* - *"* *I think that sometimes the pain can be so great that is difficult to concentrate and think clearly. So it is relevant because it's linked. if you are in that amount of pain, sometimes it's difficult to think about something else. It's difficult to even remember things because your mind just keep telling you, look this is hurt so much. Nothing else can come into play because that's all I can think about."* *(09 F 60-)* - *“I think that would definitely be really relevant to me. Not so much thinking because my thinking can get a little haywire if I'm getting anxiety spirals. but in terms of memory and stuff, if I'm particularly fatigued, I can kind of lose my track mid-sentence and things like that. usually concentrating, not so much. I tend to be someone that can be pretty focused and sometimes able to do, a couple of things at once and that kind of thing.” (07 F 60-)* | - *R1 “I feel none of the time.” (06 F 60+)* |  |
| Anxious | - *“I look up some things and I think, should I go to the doctor and say this and say that and you feel like I don't want to waste the doctor's time and trying to… you get anxious about what you should or shouldn't do..” (05 F 60+)* - *"So what comes to my mind is my general anxiety. I am an anxious person. But also what comes to my mind is how the pain makes me anxious. Sometimes I'm anxious because I know that even though it's getting late and I'm going to bed, but I'm not going to rest. I know I'm going to wake up the next morning with so much pain. And that generate a bit of anxiety because you feel like, when am I going to get rest, when can I actually rest and feel well again." (09 F 60-)* | - *R3 (previously relevant) “No, not anymore. I mean I control my emotions well now so anxiety is a thing of the past.” (01 M 60+)* - *R3 (previously relevant) "* *no I don't really.. I had a period when I was in the police where I suffered from anxiety and I was given citalopram medication to ease that...that was dealt with and basically I very rarely feel anxious." (02 M 60+)* - *“"In the last seven days, did you feel anxious? I was going to say none of the time, but the council tax bill came in the other day. I think bill time tends to make you feel a little bit anxious. but no, I tend not to feel anxious at all...so nowadays, no, I'm not anxious." (03 M 60+)* |  |
| Sad/depressed | - *“I think about how it does bring a depression and a sad side to it because you feel like you're constantly pain. You also feel sometimes like a burden because you can't do the things you wish you did. But the depression as well is linked to chronic illnesses because you keep thinking… I'm quite young. I'm only 35 and if I start thinking about what the future will bring, that brings you down quite a lot because you think if I cant do that at [18-40], what am I be able to do when I'm 60? “(09 F 60-)* - *"I guess for me it's more thinking about the things I used to be able to do and that obviously makes that feeling like that worse. feeling reliant on other people makes me feel more depressed. Not being able to do things for myself when I'm only like [18-40 years] is a bit depressing." (A12 F 60-)* | - *R1 “not at all…for people that have illness or ailments ...it's very relevant” (02 M 60+)* - *R1 "…but generally speaking, the answer is probably none of the time. I'm quite happy. And I try not to be sad or depressed about anything. life's very short. so my answer would be no" (03 M 60+)* - *R3 (previous relevance): “not anymore. Again, fortunately, I came through that episode and I'm very stable now in terms of my feelings. I mean, I think we all to a certain extent we all have the odd down day and I think, that's natural, but I'm nowhere near where I was when I was depressed before.” (01 M 60+)* - *R3 (previous relevance): “At the moment, no, I cope with it. It's a case of accepting what you've got, so there's no point in getting depressed about it. yeah, it can make you sad, but it is what it is. when you've had it for so long, you just live with it. I would probably say there none of the time at the moment.” (05 F 60+)* - *R3 (previous relevance): "everybody's had depression at some point in their life, haven't they? I have. but not in the last seven days. For me, not at all." (06 F 60+)* | *"For me, I'd feel like it's sort of putting me in a box because… I'd feel like if the question just said, "Have you felt sad?" I would feel like they're minimizing it a bit. But when they put it next to depression, I think, but that's quite a broad difference between those two things. and so, for me, I think it wouldn't be hugely relevant because I have low dips, but nothing persistent." (07 F 60-)* |
| Lacked control | - *“It would come as perhaps a little of the time because if you've got things to do and regardless of how you're feeling sometimes you have to do it. So you can't always let your illness dictate what you can and can't do.” (05 F 60+)* - *"I do feel like I lack control over my day-to-day life because I want to do more but I'm not able to do more. So I want to exercise more but I can't do it. I want to be able to work a full-time job, but I'm also not able to do it. So, that is what I'm thinking when I see the question." (09 F 60-)* | - *R1 “I can control how I go about my day-to-day life and I said no I don't lack any control in my life. I think I have total control.” (01 M 60+)* - *R1 “relevant to me? No. not specifically, but I can see why it could be.” (02 M 60+)* - *R1 “…feel lack of control. No.” (03 M 60+)* - *R1 “I'm not sure this one would be relevant to me because I still feel like I would be the one making the decision to solve the issue." (08 F 60-)* - *R3 (previous relevance): “I'm going to say none of the time. I think I've got a lot of agency in my life. covid I found impossible because all of a sudden, I didn't have it, that was taken away and that was really really difficult for me. But mostly… all the time. So, I don't feel I lack control at all.” (06 F 60+)* |  |
| Pain | - *“You get pain all over your body. So for instance, I can feel the pain in my arms at the moment. I feel a pain in my chest and my stomach. So, for example, when putting washing out on the line, if I have to hold my hands up, my arms up.. can only put a couple of things up on the line and it hurts. So yes, that physical pain and then walking, your feet, your legs, your thighs and then I also have IBS. I get a lot of physical pain in my stomach as well.” (05 F 60+)* - *moderate to severe I would say. Some days I feel as though I just get on with it and then other days but I feel as though I can't. So it will be moderate to severe. To a certain extent, I feel as though if I just carry on it's going to make it worse. But you've got to get up and get on with stuff." (10 F 60-)* | - *R1 "* *Not really for my health condition because it's not so much pain as it is just struggling to breathe but it's not painful. So it's not really relevant for me." (12 F 60-)* |  |

*Each participant is represented by a participant ID based on Interviewer initials (VG/AM), interview number, sex (F/M), age group (1839, 4059, 60+), and whether they are also a carer (C) e.g. VG04 M1839_C*

*Where multiple quotes demonstrate relevance only two are shown; for concerns with relevance all cases are shown.*

## Table S4a: Summary of the themes for perceived difference between version of the item Get around: ‘no example’ or ‘used’ or ‘normally use’

| **Preference** | **Theme/justification** | **First version shown to participant** |
| --- | --- | --- |
| They are different (n=5) | - ‘No example’ vs ‘normally use’/’used’: difference in the aid as a baseline; ‘normally use’ vs ‘used’: difference in acute or chronic use of aids (n=5) | - ‘no example’ (n=3) - ‘used’ (n=2) |
| Two versions are the same (n=5) | - ‘used an aid’ is similar to ‘normally use’ [considers the aid as a baseline], but ‘no example’ is different [asking about the use of aids] (n=4) | - ‘no example’ (n=3) - ‘normally use’ (n=1) |
|  | - ‘normally use’ and ‘no example’ are the same [considers the aid as a baseline], but different from ‘used’ [asking about the use of aids] (n=1) | - ‘normally use’ (n=1) |
| They are the same (n=2) |  | - ‘used’ (n=1) - ‘normally use’ (n=1) |

## Table S4b: Themes and quotes supporting preferences for modification of the item Get around: ‘no example’ or ‘used’ or ‘normally use’

| **Preference** | **Theme** | **First version shown to participant** | **Quote** |
| --- | --- | --- | --- |
| Prefer no example (n=3) | **Readability** ‘used’ is considered less formal language (n=2) | - ‘used’ (n=1) - ‘normally use’ (n=1) | - *"I actually think that the 2a [‘no example’] is better because thinking about people I know…like around where I live, quite a lot of elderly people, the shorter the question, the better to be honest… they're going to get lost in that sentence and they're going to go... I haven't got a wheelchair, no difficulty… this being despite the fact they can barely walk. So I would say the simpler version is a lot better version and stuff like a walking frame, walker if you normally use it, add in… I think that's taken for granted in the second question." (06 F 60+)* - *"2A ‘[no example’] has got less writing. so for me, because of my brain fog, sometimes I'd probably read that one better because if I read the other one, I think my mind would get distracted or I'd miss a line or two and skip it. So, I think 2A [‘no example’] is better for me because it just gets to the point." (11 F 60-)* |
|  | **Weak preference** ‘no example’ is a personal preference (n=1) | - ‘used’ (n=1) | - *"for me the middle ones [‘no example’] the only one that I need is. I don't use mobility aid so I don't need to do that comparison." (05 F 60+)* |
| Prefer ‘used’ (n=5) | **Appropriate** 'used' is inclusive of acute and chronic aid users (n=3) | - ‘no example’ (n=2) - ‘normally use’ (n=1) | - *"One says if you normally use a mobility aid such as a wheelchair. The other says if you used and some people might not want to admit they normally use it. it might be just the one off time that they've used it." (03 M 60+)* - *"Obviously it seems to be mostly talking about the physical side of things. But I do think it covers people with long-term and temporary physical barriers, which I think is quite good wording because that often isn't the case with things. So I think that kind of… it encompasses more conditions temporary and long-term." (07 F 60-)* - *"it allows for people to answer in any situation. so people that might have used a mobility aid but only for the week two weeks or whatever. so I think somebody who doesn't use a mobility aid can answer question 2B ['used'] and I also think that somebody who permanently uses a mobility aid can answer to be as well." (08 F 60-)* |
|  | **Readability** ‘used’ is considered less formal language (n=1) | - ‘normally use’ (n=1) | - "2b ['used'] is better...because it's less wordy and also it feels more… not like a job interview question." (04 F 60+) |
|  | **Appropriate** ‘used’ is better aligned with recall period (n=1) | - ‘used’ (n=1) | - *"I think the first one ['used'] because it seems more relevant to the specific 7-day period that you're asking about...to me, it would just mean if you've used a mobility aid in the last seven days like take that into consideration as opposed to normally using it out with" (12 F 60-)* |
| Prefer ‘normally use’ (n=3) | **Appropriate** 'normally use' is inclusive of acute and chronic aid users (n=2) | - ‘no example’ (n=2) | - *"I would say I would prefer the second way ['normally use'] of asking as opposed to this one [‘no example’]. I think that goes hand in hand with whether you use it or whether you are still using it….Because it's inclusive of people that have a real inability to walk unaided.” (01 M 60+)* - *"it's very rare that people use mobility aids acutely. It's not mostly people if they have to use, it they stick with it for a while. Although there's cases as I said. I think the normally… saying normally makes more sense as well because that the normality for that person is to have that mobility aid whilst the other one is acutely and is also not making just based on everything that that person do constantly. So, I think the 2a ['normally use'] it would fit a bigger amount of people" (09 F 60-)* |
|  | **Distressing** ‘normally use’ is less emotive (n=1) | - ‘no example’ (n=1) | - *"The way its worded, it makes me feel as though it's acceptable to use a mobility and I'm okay using it. It's not anything to be ashamed of." (10 F 60-)* |
| Prefer a combination of examples (n=1) | Prefer combining ‘used’ and ‘normally use’ (n=1) | - ‘no example’ (n=1) | - *“I'm hoping you're going to bring up 2C that says “if you used or normally use” because that covers all the angles. As I say, I've just got this belief that the more you tell people the more you'll get out of it...I just feel that if you had an amalgamation of the two 2 A and 2B, it would be perfect.” (02 M 60+)* |

‘Appropriateness’ covered views that the item, response option or layout fit the context better (e.g. a medical context), or had a more relevant scope or improved flow in relation to other items or was more accurate. ‘Readability’ referred to issues related to simplicity and the ease of reading. ‘Distressing or unpleasantness’ related to wording that caused negative emotions.

## Table S5a: Summary of themes for perceived differences for scale of the item Concentrating/Thinking clearly: Frequency scale vs Difficulty scale

| **Preference** | **Theme/justification** |
| --- | --- |
| They are different (n=7) | - Impact of severity scale may not align with frequency (n=6) - Difficulty scale is considered more impactful word (n=1) |
| They are the same (n=5) |  |

## Table S5b: Summary of themes and quotes on preferences for response options ‘moderate’ vs ‘some’

| **Preference** | **Theme/justification** | **Quotes** |
| --- | --- | --- |
| Prefer difficulty scale (n=6) | **Appropriate** difficulty considered a more important measure (n=3) | - *"5a [difficulty scale], the second one because you are restricted. it doesn't matter how long you're restricted when you're suffering from that problem." (01 M 60+)* - *"So something that only happens a little at a time you could maybe label as a lot of difficulty or moderate difficulty. So I think in some ways having the difficulty scale is perhaps a little more clear" (07 F 60-)* - *"It's an assumption that it's going to be difficult anyway with having health conditions. There’s going to be a certain degree of difficulty in having to deal with health conditions. So the acknowledgement that, yeah it’s fine to mention that it will be difficult." (10 F 60-)* |
|  | **Readability** difficulty considered easier to answer (n= 3) | - *"I think if push came to shove, I would go for no difficulty...it seems to flow a bit better for me when I'm reading it" (02 M 60+)* - *"I think you're asking them to answer the question rather than to see about times. The idea of having the time in the answer may well confuse people a little bit. I think saying difficulty, no difficulty, whatever is probably better." (03 M 60+)* - *"you've referred to the key word in the question with difficulty. so it keeps your focus on that." (08 F 60-)* |
| Prefer frequency scale (n=5) | **Appropriate** difficulty considered a more important measure (n=1) | - *"if I did the second one [difficulty scale] and said slight difficulty, then the person would think, she's not that bad so it's okay, but if I said sometimes, then that might make a difference to how they think then because that would perhaps need addressing then if it's sometimes because it's more than just a little of the time." (05 F 60+)* |
|  | **Readability** difficulty considered easier to answer (n= 4) | - *"it's simpler words and you may not have somebody who can even read very well." (04 F 60+)* - *"I think that's how people think. People think, some of the time, I can't remember my children's names. They don't think, I've got moderate difficulty in remembering this that or the other, do they? that's kind of more like their internal dialogue. Number five [frequency scale], I think." (06 F 60+)* - *"It's just a little bit more simple, like slight difficulty, moderate difficulty. whereas the other one is a little of the time, sometimes, often. I don't know it's probably just silly but it’s just for me, more relatable because it's just like sometimes, yeah, or a little of the time, yeah. So just a bit more easier to read kind of thing.” (11 F 60-)* - *"I would probably prefer five [frequency scale] because it would probably be easier for me to quantify how often of the time I felt like that as opposed to how difficult it has been. But that's probably just personal preference." (12 F 60-)* |
|  |  |  |
| Prefer both options (n=1) | Prefers separate questions depending on intended outcome of question (n=1) | - *"I think actually depends on what answer do you want. do you want about the frequency or do you want about how the difficult impacts you? So it depends of what the answer means, is valid to you knowing if it's frequently or not or how difficult it is. Maybe I would have both questions if I'm honest" (09 F 60-)* |

‘Appropriateness’ covered views that the item, response option or layout fit the context better (e.g. a medical context), or had a more relevant scope or improved flow in relation to other items or was more accurate. ‘Readability’ referred to issues related to simplicity and the ease of reading. ‘Distressing or unpleasantness’ related to wording that caused negative emotions.

## Table S6 Quotes for comprehensiveness (missing items) of EQ-HWB-9

| **Nothing missing** | **Missing items** |
| --- | --- |
| - *"No, nothing jumps out at me." (01 M 60+)* - *"I don't think so. I think for me, this would probably cover everything." (12 F 60-)* | - *"I would suggest one thing and that would be “do you feel you've had sufficient and adequate medical support?" (02 M 60+)* - *"I suppose the one question that I would consider relevant to me is, have you remembered over the last seven days to take your medication? And I think that's quite important. But perhaps rather than seven days again, did you take your medication yesterday?" (03 M 60+)* - *"have a box for additional information or something. And in there they could put, for example, how long have you been affected in this way? is there anything that makes it worse? Have you sought professional advice? Are you having any support currently? just to give them examples and they could… so just a free text box and they could just complete that." (04 F 60+)* - *"if I can be specific about some of the questions, then it'll give them a more detailed idea of what I'm actually going through...having space even if it's perhaps just a space at the end, not necessarily after every question, but a space at the end saying, is there anything you'd like to add on that…something along the lines of, is there anyone that lives with you or comes in that helps you with what you're doing? because it would be different for somebody who lives on their own, has nobody coming in as compared to somebody who's got somebody there to help them" (05 F 60+)* - *"I mean part of it is anxiety about is it going to get worse or is it fine, it's going to be okay, it's all been treated, but I don't know whether that's really what you're getting at. So, I mean if you just want to look at the impact of the health condition" (06 F 60+)* - *"I think about some of the other health conditions that I've got though as well and I wonder if something like “how well are you managing your health conditions” could be an interesting one" (07 F 60-)* - *"you've talked about day-to-day activities, but possibly things that you're responsible for...responsibilities implies a different level of pressure. Day-to-day activities, it's not going to hurt anybody if somebody doesn't have a shower one day. It's not going to hurt at somebody if you have a sandwich rather than a cooked meal for one day. But a responsibility, if you've got a responsibility, it's an imperative" (08 F 60-)* - *"I would ask about generally how do you feel mentally wise? So I know we ask about anxiety and about as depression and thinking clear but maybe ask how in general you feel like your mental health is impacted or maybe question if your experience is related to chronic pain or chronic illness that are not mental health related, how those impact your mental self." (09 F 60-)* - *"Something based around what others who are around me.. their interpretation of how my health conditions affect me. So something based on, if my husband could help in any way or my daughter.. sort of what do they feel about? a more direct question to them." (10 F 60-)* - *"So I don't know if you wanted to kind of, ask how other people are getting treated by their doctors. Are they listened to?" (11 F 60-)* |

*Where multiple quotes demonstrate comprehensiveness only two are shown; for concerns with comprehensiveness all cases are shown.*

## Table S7 Quotes for comprehensiveness (redundant) of EQ-HWB-9

| **Nothing redundant** | **Redundant / Overlap** |
| --- | --- |
| - *"I think it's quite comprehensive and I think it's very relevant to older people.” (03 M 60+)* | - *"you could say anxious, sad, depressed. Could you put that into the one question? Did you feel anxious, sad or depressed? Because it's all psychological, possibly that. Maybe condense that into one." (02 M 60+)* - *"that the feelings questions… lonely, sad or depressed, and possibly lacking control. They're kind of split out in different places. I don't know whether there'd be any reason to put them together" (06 F 60+)* |

## Table S8 Quotes for additional commentary of the EQ-HWB-9

| **Recall period** | **Quotes** |
| --- | --- |
| Recall period correct (n=2) | - *"As I said before, it's just not every day is the same as the others. So, when it goes over the last seven days, that gives a better idea of how you're getting on with your week cuz you could go into the doctors and be walking in doubled up, but the next day not feel so bad or vice versa. So, having it over the last seven days is much better." (05 F 60+)* - *"So I think the past seven days is good because it rules out, I bought a house last year and I was a bit anxious. Was it going to go through or that sort of thing. it is like here and now are you anxious?" (06 F 60+)* |
| Recall period problems (n=2) | - *RC1: "I might have had severe pain at some point in the last seven days. So the only question I would have is do they mean as an overall or what the peak has been?" (07 F 60-)* - *RC2: “I think they would probably be more accurate if you asked about yesterday. if you asked me what I did last Saturday, I couldn't tell you. I can't remember. and when you retire, and I assume that the majority of these questions, this questionnaire is going to be to older people… when you think older people haven't got any… what's the word… focus point. for a lot of people, every day is the same. so if you're asking how people are last Monday, they probably wouldn't be able to differentiate between Monday, Tuesday, or Wednesday. Whereas, if they asked about yesterday, it would be a lot more accurate.” (03 M 60+)* |
| **Response options** | **Quotes** |
| Issues with response options (n=3) | - *"No difficulty, slightly difficult, moderately difficult, a lot of difficulty, unable. Unable to what? I don't know quite where unable fits in. I could understand a lot of difficulty, moderately difficult, slightly difficulty, but unable. I'm not sure about that word there...unable to what? unable to do anything? but if you're unable to do anything I suppose that's the day. you stay in. Don't bother again." (03 M 60+)* - *"I don't know whether people would answer a little of the time or sometimes because there's not really a lot of difference, is there? a little of the time is really occasionally, isn't it? Or I suppose that's the same as sometimes. or every now and then, but that's a bit the same as sometimes. "I don't understand why have is in bold because have is not…. the bit that should be in bold is just “doing day-to-day activities” and again the second one… the bit should be is just “getting around inside or outside” because the have is a part of the previous introductory phrase. Did you feel exhausted? Yeah. Did you feel lonely? again I'd say the same in five. five is slightly different because that have does belong there. The structure of that sentence. Sorry, I've got an English degree. Did you have difficulty, whereas it's not, did you have getting? did you have doing?" (06 F 60+)* - *"I think it's interesting that some of them are measuring in difficulty and some of them measuring in time, which I hadn't noticed before. and I guess the interesting thing is with questions like exhausted and with sad or depressed because I guess something with depressed, I guess you would be having a measure of time but also it's the impact of that as well. but something like exhaustion, I think you might have had a day where you were completely exhausted and couldn't do anything. So that day would have had a very high impact. So the difficulty impact would have been a lot higher. So I think with questions like that, it would be a little tricky to answer because I'd think, okay I was recovering from a horrific fatigue crash. I had a day where I couldn't do anything at all. that day was only one day of the week though but its impact did cause a lot of difficulty almost unable to do anything. So I feel like it would be difficult to measure that. it's some of them I think because there's a mix of the time and the difficulty measure it almost feels like both measures should be on some of them to kind of really demonstrate the impact" (07 F 60-)* |
| **Instructions** | **Quotes** |
| Positive response to instructions (n=2) | - *"so, yes, please choose one answer. yeah, that's a good point. You better set it up so they can only choose one." (06 F 60+)* - *"I think that's quite an important difference to put in there because you might just manage to do something, but it may be nowhere near how you would want to have something done. So, I think that is quite a good thing to include. But feelings… is an interesting one because that's not something that often comes up. Things are usually much more black and white than that. And I think feelings are something that I always feel just gets swept to the side and not really considered. So that's an interesting word to see there." (07 F 60-)* |
| Negative response to instructions (n=2) | - *"the one thing I have trouble thinking about is “have them done for you or as you want it”. I don't really understand that, what you're actually wanting." (01 M 60+)* - *"I think those instructions are a bit repetitive actually and the second sentence restates the first sentence and I think, certainly dealing with the public, as I have had to in my life, you need it as simple as possible. So that first sentence absolutely fine but the second sentence, think about your health, feelings and… how can you do it without thinking about your health and feelings. I mean it's one of those typical things where you just think…. you're not going to say don't think about your health and feelings just tick any old box, are you?...to do your activities or have them done for you as you wanted. I think that's very complicated. I really think a lot of people would not understand what that meant to be honest. whether you were able to do your activities with or without help. I'd say it's something like that. Or do the activities you want with or without help or whether or not you needed help, something like that. So, I think those instructions do need to be simplified actually.” (06 F 60+)* |
| **General comments** | **Quotes** |
| Positive response (n=6) | - *"one question in it, I've never had before and it's extremely relevant to me and that is question number five but I've never had that asked of me. “do you have difficulty functioning thinking clearly” and I think that's a very important point to make" (01 M 60+)* - *"it's very well set out. It's unambiguous. It's very easy to read. I don't think it's difficult to understand...the questions are fair and the scale of how people are feeling is quite well put down as well." (02 M 60+)* - *"I think it's quite comprehensive and I think it's very relevant to older people." (03 M 60+)* - *"it does look as if all unnecessary language has been taken out, which is good. And I like the bold type at the end, and that's consistent across the questions. And I like that." (08 F 60-)* - *"I think its really well worded. really is a nice questionnaire that it seems like it covers everything basically, but without going in too deep and without making it too hard for you to do it or wanting to postpone because it's so long." (09 F 60-)* - *"And I think they are all kind of relatable because you do feel lonely. You do get exhausted. So I think every question there, needs to be there for me. So a lot of people don't ask about if you're lonely and whatever. They just kind of go on the symptoms. But it is very lonely kind of being ill all the time cuz you're not fun...it's also very expensive being sick." (11 F 60-)* |
